# Reported incidence and risk factors of childhood pneumonia in India: a community-based cross-sectional study

**DOI:** 10.1186/s12889-018-5996-2

**Published:** 2018-09-11

**Authors:** Jayashree Gothankar, Prakash Doke, Girish Dhumale, Prasad Pore, Sanjay Lalwani, Sanjay Quraishi, Sujata Murarkar, Reshma Patil, Vivek Waghachavare, Randhir Dhobale, Kirti Rasote, Sonali Palkar, Nandini Malshe

**Affiliations:** 1 Department of Community Medicine, Bharati Vidyapeeth Deemed to be University Medical College, Off Pune Satara Road, Pune, 411043 India; 2 Department of Community Medicine, Bharati Vidyapeeth Deemed to be University Medical College, Sangli, India; 3 Department of Pediatrics, Bharati Vidyapeeth Deemed to be University Medical College, Pune, India

**Keywords:** Overcrowding, Unclean fuel, Inadequate ventilation, Exclusive breastfeeding, Immunization status, Nutritional status, Hand hygiene

## Abstract

**Background:**

Pneumonia is responsible for high morbidity and mortality amongst children under five year of age. India accounts for one-third of the total WHO South East Asia burden of under-five mortality. There is a paucity of epidemiological studies indicating the true burden of pneumonia. Identification of the risk factors associated with pneumonia will help to effectively plan and implement the preventive measures for its reduction.

**Methods:**

It was a descriptive cross-sectional study conducted in 16 randomly selected clusters in two districts of Maharashtra state, India. All mothers of under-five children in the selected clusters were included. A validated pretested interview schedule was filled by trained field supervisors through the house to house visits.WHO definition was used to define and classify clinical pneumonia. Height and weight of children were taken as per standard guidelines. Quality checks for data collection were done by the site investigators and critical and noncritical fields in the questionnaire were monitored during data entry. For continuous variables mean and SD were calculated. Chi-square test was applied to determine the association between the variables. Level of significance was considered at 0.05.

**Results:**

There were 3671 under five-year children, 2929 mothers in 10,929 households.Unclean fuel usage was found in 15.1% of households. Mean birth weight was 2.6 kg (SD;0.61). Exclusive breastfeeding till 6 months of age was practiced by 46% of mothers. Reported incidence of ARI was 0.49 per child per month and the reported incidence of pneumonia was 0.075 per child per year. It was not associated with any of the housing environment factors (*p* > 0.05) but was found to be associated with partial immunization (*p* < 0.05). Poor practices related to child feeding, hand hygiene and poor knowledge related to signs and symptoms of pneumonia amongst mother were found.

**Conclusions:**

Very low incidence of pneumonia was observed in Pune and Sangli districts of Maharashtra. Partial immunization emerged as a most important risk factor. Reasons for low incidence and lack of association of pneumonia with known risk factors may be a better literacy rate among mothers and better immunization coverage.

**Trial registration:**

Registration number of the trial- CTRI/2017/12/010881; date of registration-14/12/2017.

## Background

Globally pneumonia is responsible for high morbidity and mortality among children under 5 years of age. The World Health Organization (WHO) has estimated an incidence of 0.37 episodes per child per year for clinical pneumonia, India accounts for 36% of the totalWHO South East Asia regional burden [1, 2]. Approximately 10 to 20% of these episodes tend to be severe [3]. In the most recent estimate of Acute Lower Respiratory Infections associated mortality in India(2014), pneumonia was held responsible for 369,000 deaths (28% of all deaths), making it the single most important killer in this age group [4]. The national level surveys of India reported the prevalence of ARI only, which was found to be 2.4–8.9% for the state of Maharashtra [5, 6].

Childhood clinical pneumonia is caused by exposure to risk factors related to the host, the environment and infection. Risk factors like lack of exclusive breastfeeding, low birth weight, under-nutrition, indoor air pollution, overcrowding and lack of measles immunization are associated with pneumonia. These risk factors are categorized as definite, likely and possible based on the evidence pointing to their role in pneumonia [1, 2].

Reduction of these risk factors is suggested as a primary strategy to protect against pneumonia. Community-Based Interventions(CBI) including mother’s education for reduction of risk factors is an important intervention measure for the long-term sustainability [7, 8]. In India, there is a lack of evidence on epidemiology and etiology of pneumonia posing as an important barrier for effective planning and implementation of preventive measures [4].

This the study was planned and conducted to identify the total episodes of ARI and pneumonia as reported by mother in the previous one month and one year respectively; to corroborate the association of risk factors with pneumonia in a large-scale community-based study.

### Objectives

1. To measure the reported incidence of ARI in last one month and reported incidence of pneumonia in the last one year among under-five children
2. To determine the association of factors like ventilation status, overcrowding, type of fuel used for cooking, indoor smoking by family members, immunization and nutritional status of children, knowledge, attitudes and practices(KAP) of the mother with pneumonia.

## Methods

### Study design

We adopted a descriptive cross-sectional study design.

### Study setting

The study was conducted in urban and rural field practice area of two medical colleges of Bharati Vidyapeeth Deemed University located in Pune and Sangli districts of Maharashtra state, India. These two districts are similar with respect to health indicators and have almost equal composite health index(Pune = 0.65& Sangli = 0.66) [9]. The total population of these four units is 1,89,504. All the slums under urban field areas are notified. The rural field practice areas have 9–16 revenue villages each under their jurisdiction. The notified slums or revenue villages under these field practices areas are hereafter referred to as clusters. There are total 45 clusters with average 250 under five children per cluster.

### Study duration

The duration of this study was about six months including three months of data collection from January to July 2015.

### Number of clusters

Considering incidence of childhood pneumonia to be 0.3 episodes per child per year, to achieve 80% power, with a confidence level of 95%, a design effect of 2 and nonresponse rate of about 15% the study required to include 16 clusters [10].

### Selection of clusters

**Fig. 1:**
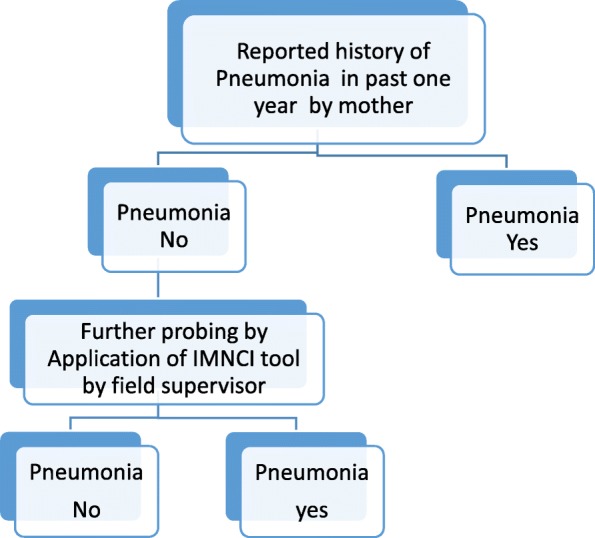
Shows the decision tree for pneumonia. In past one year, if any of the respiratory illness was not reported by mother as pneumonia, then further probing was done by the field supervisor using IMNCI tool . An episode of pneumonia was labelled if the use of IMNCI tool indicated so

The total clusters under the field practice area were first stratified district wise and then into the urban and rural area, each area was further divided geographically into two regions (East-West or Primary PHC wise). From these regions, two clusters each were randomly selected using random numbers generated by Micro soft Excel. A total of 16 clusters were thus selected. The flow chart of the study area and selected clusters is depicted in Fig. 1.

Study participants: All mothers of under five-year children in the selected clusters were included in the study.

### Definition of study variables

1. Type of family: Was divided into two groups; nuclear family consists of the husband, wife and their unmarried children staying together, joint family included all other families including three generation family as well as extended family. Households where its occupants were not blood-related i.e. household occupied by nonblood related members were not considered as family and not included in the study.
2. Socioeconomic status of the family: Under tricolor ration card scheme in the state of Maharashtra, the families had been already grouped as per the color of ration card. Yellow, orange and white colored ration card are distributed to families with income up to Rs. 15,000, between Rs. 15,000 to 1lakh and more than Rs.1 lakh per annum respectively [11].
3. Ventilation: Inadequate ventilation included households with less than 50 sq.ft. floor area per person irrespective of the presence of fan or 50–100 sq.ft. of floor area per person without a fan. Adequate ventilation included households with 50–100 sq.ft. area per person with a fan or more than 100 sq.ft. per person irrespective of the presence of a fan.
4. Overcrowding: Accepted standards of number of per person per room were used. If the number of persons per room is more than these criteria, overcrowding was considered to be existing [12].
5. Type of fuel used for cooking for most of the days of the week was classified as clean and unclean fuel. Clean fuel included Liquefied Petroleum Gas (LPG) or Electric Shegdi; unclean fuel included biomass, coal Shegdi, stove with kerosene [13].
6. Educational status including Literacy status: It was applicable to individuals above the age of 7 years. Illiterate is a person who cannot read, write and understand any one language [13]. Educational status was classified according to years of schooling.
7. Birth weight of children: As reported by mothers or found from records
8. Immunization status: Children who received all vaccines as per the national immunization schedule, till one year of age were labeled as fully immunized. Children who did not receive at least one vaccine were labeled as partially immunized and the children who did not receive a single vaccine were labeled as non immunized. Children whose records were not available were excluded from the analysis.
9. Height and weight of children: were taken as per standard guidelines. Nutritional assessment was done as per WHO guidelines [14, 15].
10. ARI: a Cough and/or difficulty in breathing irrespective of fever
11. Pneumonia: WHO guidelines for clinical pneumonia were used. Guidelines classify pneumonia as follows if the mother reported a history of a cough and/ or difficult breathing and fast breathing that episode was labeled as pneumonia. History of a cough and/ or difficult breathing and chest indrawing irrespective of the rate of breathing was labeled as severe pneumonia. While the very severe disease was labeled when the child had an inability to drink, persistent vomiting, convulsions, lethargic or unconscious, stridor in a calm child or severe malnutrition [16].
12. Cause of death in under five children: For each death reported, the cause of death as reported by mother was considered.

### Research tool

The interview schedule was prepared in English, validated by experts, translated in Marathi (local language) and then back-translated into English. The translation was certified by a subject and language expert. A unique identification code was allotted to the household, mothers of under-five children and the children. The validated and pretested interview schedule consisted of closed and semi-open-ended questions and had following sections:

1. Information related to household
2. Details of 0–5-year-old children
3. Details of children aged 0–1 year
4. Immunisation status of children aged 12–23 months
5. The section on KAP of mothers related to pneumonia.

The study was started after the approval of the ethics committee.

### Data collection

The data was collected by eight project appointed field supervisors (FS) who were well versed in the local language (Marathi). Activities of each field supervisor were monitored by a designated site investigator (SI). Site investigators were doctors and faculty from two medical colleges. The data were collected simultaneously in all clusters through the house to house visits. All Accredited Social Health Activist (ASHA) in three areas and Anganwadi workers (AWW) in Pune urban area from the selected clusters were identified and their cooperation was sought during the data collection.

All families in the selected cluster residing for a period of more than six months before the data collection were included. Some houses which were locked, an inquiry about the absence of the family was conducted from neighbors. When inquiry indicated permanent migration, those families were excluded and the second visit was given to remaining households during the evening or early morning or on weekend. Those households which were found to be locked on the second visit also were excluded from the study. A written informed consent was obtained from all mothers. Information pertaining to the first section was obtained from all households. If there was a child under five year of age(i.e. not completed 5 years of age) in the family, further sections of the tool were filled by interviewing the mother. Ventilation of household was assessed after obtaining information about total floor area of the house in square feet excluding the bathroom and water closet area. This was based on information provided by the head of the family or mother of under-five children or as judged by the interviewer. Use of fan implies an increase in the air current, so we considered the presence of fan as a factor affecting ventilation of the households. Since the majority of the households used a fan in their households, it was considered for ventilation status criteria. The history of immunization for children between 12 and 23 months of age was enquired with mothers and confirmed through immunization cards. However, the children whose immunization cards were available only were considered for analysis. Information on vaccines apart forms national immunization schedule was also collected. Weight and length/height of all children was measured and recorded. In illnesses of respiratory systems, mothers were asked about seeking treatment and diagnosis conveyed by the doctor or health care provider. An episode of pneumonia was recorded if it was conveyed to her by the treating doctor or health care provider. In case there was no such evidence, further probing was done by FS to ascertain the pneumonia episode based on the signs and symptoms reported by mother.Thereafter the trained FS labeled the episode as pneumonia or no pneumonia based on IMNCI classification (Fig. 1). Information about exclusive breastfeeding was collected for children between 6 months to one year of age only.

Quality checks for data collection were done through supervisory visits by SI. The data collected by FS were cross-checked by SI to ensure completeness and accuracy prior to data entry.

### Data management

All forms collected were entered into the software database simultaneously. Critical fields in the tool were identified as a proxy to the completeness and accuracy in the form. Both critical and noncritical fields were monitored. For critical and non-critical data, discrepancies up to 0.1% and 1% respectively were considered acceptable. Alternate forms were physically cross-checked for discrepancies related to data entry. Data clarification report was shared with field supervisors and site investigators for any corrections if needed. Missing data were not imputed because of low occurrence and complete cases were analyzed. The data was cleaned, analysed and the report was prepared.

### Data analysis

Data were coded and analyzed using SPSS version 20. For categorical variables, data were presented as number and percentage. For continuous variables, mean and SD were calculated. Chi-square test was used to find an association between variables of urban and rural clusters. Level of significance was two-tailed and considered at 0.05.

## Results

### General information

There were 12,344 houses in selected 16 clusters from two districts, out of which 1415 houses were not included as they were locked on two consecutive visits or the members were uncooperative or families were living for less than last 6 months. Thus the total household covered were 10,929 with 48,908 population. The total number of households under various sections described below may vary depending on the missing information. Out of the total, 2799 households had children under five year age.

Majority of the households in the study clusters were Hindu (86.6%), 5.2% were Muslim, 5.0% were Buddhist, other religion included Jains and Sikhs. Households having other religion were more in rural than in urban clusters (*p* < 0.05). Out of total households, 54.1% belonged to nuclear families. Nuclear family were more in urban clusters (58%) than rural clusters (50.3%) (*p* < 0.05). The average family size was 4.48 with (SD;1.9). The literacy rate among the head of the family was found to be 67.0%, it was similar in rural and urban (*p* > 0.05). Out of total households, 89.3% of the total families had income up to Rs. one lakh per annum, they were 90% in urban clusters and 2.0% less in rural clusters (*p* < 0.05).

### Housing environment

Overcrowding was existing in 43% of households. In urban clusters 49.3% households and in rural clusters 36.8% of households were overcrowded(*p* < 0.05). Majority i.e. 94.4% of households were inadequately ventilated. More households in urban clusters were inadequately ventilated than rural clusters (*p* < 0.05). Prevalence of indoor smoking was found to be 3.2% and 2.1% in urban and rural clusters respectively (*p* < 0.05). Out of total households, 15.1% used unclean fuel. The use of unclean fuel is more in rural clusters (24.0%) as compared to urban clusters (6.0%) (*p* < 0.05).Out of total households, 5.0% were having mud/earthen flooring.

### Information about under-five children

There were 2929 mothers having under-five children. There were 5.4% illiterate mothers, 9.1% were educated in middle school and 56.5% were educated up to high school and the remaining 18.0% were educated 12^th^or above including graduates. The response of mother as don’t know or don’t remember was very less. There were 3671 under five children constituting 7.5% of the total population. The child sex ratio was (number of girls per 1000 boys) was found to be 893. There were 752 infants; out of which for 116 infants either mothers could not remember or did not have a record of birth weight, for remaining infants the mean birth weight was 2.6 kg (SD;0.61).

Among infants, 92.3% received colostrum as the first feed. Higher proportion i.e. 98.0% of children in rural clusters received colostrum than children (86.0%) in urban clusters (*p* < 0.05). Remaining children received honey, sugar water or milk powder as the first feed.

Around 46.0% of the children between 6 and 12 months were exclusively breastfeeding (EBF) till six months of age. About 78.0% of children in rural clusters received EBF till 6 months of age compared to 15.4% of children in urban clusters. (*p* < 0.05). Out of total 768 children between 12 to 23 months of age, 639 children were having records of primary immunization. Out of 639, the overall percentage of fully immunized children was 605 (94.6%). It was slightly lower for urban clusters (91.5%) compared to rural clusters (97.0%) (*p* < 0.05). Majority of the children i.e. 96.0% received measles immunization. Out of total children, 9.4%, 12.4% received newer vaccines like PCV and Hib respectively (Table 1).

**Table 1:** Nutritional status of children

Nutritional status (weight for height)	Urban No.(%)	Rural No.(%)	Total No.(%)
Severely wasted (< −3 SD)	323(18.4)	286(15.8)	609(17.1)
Wasted(<−2 SD)	176(10)	214(11.8)	390(10.9)
Normal	1097(62.5)	1129(62.5)	2226(62.5)
Overweight/obese	160(9.1)	69(9.9)	338(9.4)
Total	1756(100)	1807(100)	3563(100)

On a weight for height parameter,17.1% and 11.1% of children respectively were wasted or severely wasted. Distribution of wasted and severely wasted children in urban and rural areas was almost similar (*p* > 0.05)

### ARI and pneumonia

The reported incidene of ARI was 0.49 per child per month i.e. on an average a child suffers from 6 episodes of ARI per year. The incidence was more in rural (0.53) than urban clusters (0.43) (*p* < 0.05) (Tables 2 and 3).

**Table 2:** Incidence of Pneumonia(1 month-5 years of age)

Area	No. of children with pneumonia/Total children	Rate per child/year
Rural	146/1782	0.13
Urban	135/1787	0.07
Total	281/3569	0.075

The pneumonia episodes per child per year were 0.075 across the study clusters for children between 1 month-5 years of age. The number of episodes was 0.13 per child per year in rural clusters and 0.07 per child per year in urban clusters. (*p* > 0.05)

**Table 3:** Age and sex distribution of pneumonia cases

Age group(Years)	Female No.(%)	Male No.(%)	Total No.(%)
0 ≤ 1	18(36.00)	32(64.00)	50(100)
1 - < 2	32(40.00)	48(60.00)	80(100)
2 - < 3	37(57.81)	27(42.19)	64(100)
≥ 3	31(35.63)	56(64.37)	87(100)
Total	118(41.99)	163(58.01)	281(100)

The incidence of pneumonia was in general higher for male children(58%) in all age group excepting 2–3 years. (*p* < 0.05)

There was no case reported of severe pneumonia as well as very severe disease. Total 3 deaths were reported due to pneumonia and an additional 11 deaths were due to other causes in one year preceding the survey. Thus the child mortality was 3.81 per 1000 children aged 0 to 4 years. The proportionate death rate for Pneumonia for one year in the study clusters was 21.42%.

### KAP of mothers

There were 2929 mothers of under-five-year-old children with a mean age of 24 years (SD;6.64). The responses are analyzed based on a number of children. Hence the number of responses is more than 2929 and less than 3671. Majority of mothers (79.0%) had correct knowledge regarding the ideal duration of exclusive breastfeeding. Fever (91.0%) followed by common cold (43.0%), pneumonia (36.0%) and diarrhea (30.0%) were commonest childhood illness perceived by the mothers.

Out of the total, 52.1% of the mothers were not aware of any symptoms of pneumonia. Only 2.0% of mothers could tell correctly all symptoms of pneumonia and 16.0% reported rapid breathing as one of the symptoms of pneumonia. While 14.0% reported chest in-drawing as one of the danger symptoms. Majority of women (77.7%) preferred private practitioner in the cluster or in the vicinity of the cluster for seeking treatment for pneumonia. Out of all mothers, 59.0% reported that pneumonia can be fatal in children. Importance of ‘Hand hygiene of mother’in the prevention of illnesses in children was acknowledged by 11.0% mothers.

In the current study, only 7.3% of mothers had correct knowledge about hand hygiene, it was significantly higher among mothers from rural clusters than urban clusters (*p* < 0.05). Maximum i.e. 819 mother perceived that hands to be washed after defecation only. About an equal number of mothers did not mention the need for hand washing at any specific occasions. Only 4 mothers perceived the need to wash hand on all four occasions however surprisingly, a larger number i.e. 139 mothers practiced washing of the hands-on all these occasions. While 849 women wash hands after defecation only.

A significant association was found between pneumonia cases and the status of immunization (Table 4). The odds of having pneumonia was higher in children who received partial immunization, (22%, 8/36) than children who received a full immunization (OR = 2.41, 95% CI; 1.06–5.49). It was found that the odds of children suffering from pneumonia is higher in mothers who had the awareness that both indoor air pollution and overcrowding are risk factors for pneumonia (OR = 0.68, 95% CI; 0.52–0.88). It was found that the odds of children suffering from pneumonia were higher in mothers who had the awareness about symptoms of Pneumonia (OR = 0.36, 95% CI; 0.26 to 0.48). It was found that the odds of a child suffering from pneumonia were higher in mothers who had the awareness about symptoms of severe Pneumonia (odds ratio = 0.27,95% CI; 0.18 to 0.40). The knowledge about risk factors was similar among mothers of urban and rural clusters. (*p* > 0.05). We believe that the episode of pneumonia in her child and subsequent interaction with the doctor or ANM might have improved mother’s knowledge. Since episode and subsequent increase in knowledge both occurred in past one year, it is appearing that the more knowledge was associated with pneumonia episodes.

**Table 4:** Association of various factors with pneumonia

Factors		Pneumonia		
		Yes	No	Total
Colour of ration card	Orange and yellow	237	2924	3161
	Others(white, no card missing info.)	44	466	510
Ventilation status(*n* = 3520)	Adequate	9	114	123
	Inadequate	263	3134	3397
Presence of overcrowding(*n* = 3651)	Yes	203	2293	2496
	No	78	1077	1155
Indoor smoking by family member (*n* = 3650)	Yes	14	117	281
	No	267	3252	3519
Type of fuel for cooking(*n* = 3655)	Clean	227	2892	3119
	unclean	54	482	536
Area(*n* = 3671)	Urban	135	1699	1834
	Rural	146	1691	1837
Sex of child	M	163	1776	1939
	F	118	1614	1732
Immunisation status*(*n* = 639)	Full	64	541	605
	Partial	8	26	34
Measles immunisation(*n* = 653)	Yes	68	561	629
	No	3	21	24
Exclusive Breast Feeding(*n* = 445)	Upto 6 months	17	189	206
	> = 6 months	14	225	239
Birth weight (*n* = 636)	> = 2.5 kg	37	400	437
	< 2.5 kg	06	193	199
Nurtitional status (*n* = 3589)	Wasting/severe wasting	82	906	988
	Normal	188	2354	2542
Awareness that malnurishment is a risk factor for pneumonia (n = 3589)	Yes	12	173	185
	No	268	3212	3480
Awareness that poor hand hygiene is a risk factor for pneumonia (*n* = 3526)	Yes	7	76	83
	No	273	3309	3582
Awareness that indoor air pollution and overcrowding are risk factors for pneumonia (n = 3589)*	Yes	192	2027	2219
	No	88	1358	1446
Handwash After at least 3 or four occasions (*n* = 3665)	Yes	21	246	267
	No	259	3139	3398
Mothers awareness about symptoms of Pneumonia (*n* = 3189)*	Yes	80	431	511
	No	200	2954	3154
Mothers awareness about symptoms of severe Pneumonia (*n* = 3189)*	Yes	43	180	183
	No	237	3205	3506

* Statistically significant; χ2 test (*p* < 0.05)

## Discussion

Pneumonia affects children irrespective of socioeconomic status; the risk of acquiring pneumonia is higher among young infants, malnourished children, non-exclusively breastfed children and those with exposure to solid fuel use [1]. There is a paucity of large-scale epidemiological studies related to pneumonia. This study is probably unique in providing information about the incidence of pneumonia and its risk factors at the community level. The data was collected by qualified and trained field supervisors and the quality checks for data collection was monitored by the site investigators from Public Health, Community Medicine and Pediatrics specialty. The demographic data and housing environment conditions obtained in the study were compared with available state or district data for compatibility and the data was reasonably similar. The yellow and orange ration card holder families are eligible for getting benefits under a scheme named Mahatma Jyotiba Phule Jan Arogya Yojana. The scheme envisages improved access of Below Poverty Line (BPL) and Above Poverty Line (APL) families (excluding White Card Holders) to quality medical care for identified specialty services requiring hospitalization for surgeries and therapies or consultations through an identified Network of health care providers. However socioeconomic status based on the ration card color category was not associated with the incidence of pneumonia.

### Housing environment

Indoor air pollution and overcrowding are some of the risk factors associated with pneumonia [2]. Overcrowding and inadequate ventilation increases interior moisture and provide a nurturing environment for mites, respiratory viruses, and molds. It is well known that they all play role in respiratory disease pathogenesis [17]. Use of unsafe fuel like crop residues, dung, wood, and coal in poorly ventilated houses may lead to accumulation of smoke in and around the home. Mothers and their young children, who spend the most time indoors are exposed to this smoke [18].

In the current study, 43% of households were overcrowded with the average family size of 4.48. The majority (94%) of households were inadequately ventilated. The percentage of a joint family in current study reports figures of 46% lesser than the Figs. (70%) reported in a national survey for Maharashtra state. The average family size in the current study is higher than that reported by the national survey for Maharashtra state figure of 3.4 [19]. Although we did not observe any association of overcrowding as well as inadequate ventilation with pneumonia. Various studies have indicated overcrowding as a significant risk factor forARI [20, 21].

Unclean fuel for cooking is used by 15.1% households in the study clusters. As the biomass fuel is easily available in the rural area, in current study its use is seen more in a rural area (24%) than in urban area (6%). State level surveys have reported 40–43% of households used unclean fuel for cooking. A household survey conducted in India found a statistically significant relationship (OR 1.3) between reported use of household biomass fuel and reported the incidence of respiratory infection in the previous week among children under five years [22]. In the current study, there was no association found between type of fuel used and pneumonia (*p* > 0.05) Other studies too have reported the association of use of biomass fuel and prevalence of ARI [19, 23].

In the current study, only 5% of households have mud or earthen flooring. Economic survey of Maharashtra 2012–13 which includes tribal areas has reported a higher percentage of households (36.1%) with a floor made up of mud. However, these findings are about 5 years old.

Exposures to environmental tobacco smoke (ETS)is consistently related to respiratory symptoms. It has been shown to adversely affect components of the defense mechanisms against infectious organisms. We were more interested in indoor smoking rather than the proportion of smoking individuals. Most of the surveys report the proportion of smoking individuals with logically higher figures like 4.7% and 18% [5, 6].

### Breastfeeding practices, nutritional and immunization status of children

Prevalence of exclusively breastfeeding(EBF) till six months of age in the current study was found to be 46%, while state-level reports of national surveys have shown the prevalence of 53–68% [5, 6]. There was no association of EBF with reported pneumonia in the current study (*p* > 0.05).

By weight for height parameters, 28% of children were wasted. These figures are lower than the state figs. [5, 6]. There was no association of wasting with pneumonia found in the current study (*p* > 0.05).

The percentage of fully immunized children between 12 and 23 months in the current study was found to be higher (94.6%) than the National level survey for Maharashtra state have reported these percentages to be 57% and 66% [5, 6]. In the current study, the rates for measles immunization is reported to be higher too i.e.96.4% compared to National surveys for Maharashtra state figures ranging of 83% and 86% [5, 6]. The current study did not include migratory and tribal population leading to higher figures of immunization coverage. In addition, these figures did not include the immunization status of 110 children (14%) with missing immunization card. The mothers of these children were negligent in preserving immunization cards and hence we feel that among these children the immunization status may not be as high as 94.6%. In a Demographic and Health Surveys (DHS) done in the Republic of Congo, Ethiopia, India, and Pakistan found that Children who were given the measles vaccine were less likely to suffer from ARI than unvaccinated children in India and Pakistan [24]. In the current study, Pneumonia was reported to be higher in a partially immunized child than in fully immunized children. (*p* < 0.05). Other studies have reported partial or lack of immunization as a significant risk factor for pneumonia [21, 25, 26]. This study also substantiated the higher risk of pneumonia among partially immunized children.

### ARI and pneumonia

Lack of access to health care and social factors are responsible for the high burden of pneumonia. Coexisting diarrhea, measles, and severe malnutrition are responsible for the severity of the disease. Low birth weight (LBW) is a major contributor to pneumonia morbidity.

In the current study the incidence of ARI for preceeding one-month is 0.49, it is slightly higher in rural clusters (0.52) than urban clusters (0.46). Other studies done in India reports the similar figures for ARI [6, 27, 28].

One study in India reports the prevalence of ARI to be 59.1% but contrarily it was higher in urban areas (63.7%) than rural areas (53.7%) [17]. A situational analysis quotes that the ARI contributes to 58.8% of total childhood morbidities [10]. However, the national level survey for Maharashtra state reports the very low prevalence of symptoms of acute respiratory infection (ARI) in the last 2 weeks preceding the survey (2.4%). These differences in prevalence might be due to the differences in socio-economic, cultural and risk factor exposure and methodology adopted in the study.

The incidence of pneumonia in the current study was 0.075 episodes per child per year. It was more in rural clusters (0.13 per child per year) than urban clusters (0.07 per child per year). The reason for this urban-rural difference might be due to the gap in the utilization of existing services, family practices leading to delay in seeking care.

In the diagnosis of pneumonia, fast breathing is a single most important sign. In the current study, pneumonia was not diagnosed by counting of breathing rate by mothers but it was based on the perception of mothers as a high rate and excessive up and down movement of the chest during breathing. None of the mothers could tell about the presence of chest indrawing when her child was having a cough and or difficult breathing. An incidence of pneumonia for India between 0.2 to 0.5 episodes per child-year were reported through a literature survey based on studies conducted approximately between the year 1990 to 2010 [3]. A community-based study in Philipines reported the incidence of pneumonia-like episodes to be 0.16 per child per year [29]. In the current study, mothers could not correctly tell about the danger signs of pneumonia. The average education of mother in the current study was to secondary schooling i.e. 7–10 years of total education.

The under-five mortality in the study was 3.7 per 1000. Although our observation is based upon small numbers it is very close to the rate of 4.7 reported for Maharashtra state in 2015 [30]. Three deaths were reported due to pneumonia, thus 8 deaths per 10,000 under five population due to pneumonia were reported in the current study, while other studies have reported 32.2 deaths per 10,000 under five population due to clinical pneumonia among year old children in India [2, 31].

### KAP of mothers

The results of the study may be interpreted in the background of the high educational status of mothers. In the current study higher number of mothers reported pneumonia as the commonest childhood disease. This is a true reflection of morbidity reported in India; among communicable disease, Acute Respiratory Infection accounts for 67% of cases [32]. Only 16% of mothers reported rapid breathing as one of the symptoms of pneumonia These findings are similar to the study done in Uttar Pradesh where the recognition of danger signs of pneumonia amongst caregivers was reported to be poor and fast breathing as an early sign was not commonly recognized while chest indrawing was recognized as a sign of serious illness [33]. National level survey for Maharashtra state reports 52% of women were aware of the danger signs of ARI [5]. Hand washing with soap and water removes pathogenic organisms from hands. If mothers wash their hands, they are less likely to transmit pathogens from their hands to their mouths and to articles handled by them.

Hence hand hygiene of the mother is crucial to prevent communicable diseases in children. The practice of hand wash among mothers was found to be poor in the current study, 19% wash their hands after defecation only. A study done in South India reported, 73.18% and 63.91% of respondent mothers respectively practiced hand washing with soap and water after defecation and after cleaning child who has defecated, and only 20.92% washed hands with soap and water before preparing food [34].

Mothers whose children suffered from pneumonia must have gathered information directly or indirectly about risk factors like overcrowding as reflected in the high odds ratio. Awareness of mother about smoking and overcrowding as a risk factor for pneumonia in children is preventing pneumonia in children. Since there was not a single case of severe pneumonia or severe disease the possibility of mothers having knowledge about severe symptoms is low (Table 4).

The current study reports a lower incidence of pneumonia than other studies. The most probable reason might be high immunization coverage. This is also substantiated by the fact that in spite of 17% of children being severely wasted the mortality due to pneumonia was minimal though undernutrition act as a risk of death due to pneumonia [35]. Secondly, on average maximum mothers have had 7–10 years of schooling i.e. almost all mothers are educated in high school. Additionally, there was minimal use of unclean fuel in the households and indoor smoking by family members was almost negligible. The health indicators of these two districts are better in the state of Maharashtra.

The proportion of households in rural clusters using unclean fuel resulted in the higher occurrence of ARI than urban clusters. Apparently, the incidence of pneumonia was also higher in rural clusters, but was not significant; probably due to small numbers. Secondly, the favorable factors like less overcrowding, less indoor smoking and better ventilation may have overruled the adverse effects of the higher use of unclean fuel in rural clusters in the occurrence of rural clusters.

#### Limitations

In the study WHO definition of clinical pneumonia was used. This method has high sensitivity but low specificity. Diagnosis of pneumonia was not based upon counting of respiratory rate. Radiological confirmation of pneumonia was not available. Pneumonia has various peaks in different seasons, this seasonal variation in pneumonia incidence could not be captured in the current study. Since chest indrawing was difficult to be reported by the mother, none of the pneumonia episodes could be reported as severe pneumonia. Since the history of pneumonia was taken from mothers there was the possibility of recall bias.

#### Recommendations

There is a need to conduct community-based epidemiological studies measuring the incidence of pneumonia in children over one full year by executing active surveillance. The frequency of these visits should not be more than a fortnight. Any frequency more than fortnight will lead to recall bias leading to underestimation of the problem [29]. There is need to create awareness regarding exclusive feeding, adequate weaning practices, hand hygiene and awareness about signs and symptoms of pneumonia amongst mothers through ASHA or Anganwadi workers.

## Conclusions

Very low incidence of pneumonia was observed in Pune and Sangli districts of Maharashtra. Partial immunization emerged as a most important risk factor. Among all studied risk factors only use of unclean fuel was high in the rural area. It may have led to the higher occurrence of ARI in rural clusters. Apparently, the incidence of pneumonia was also higher in rural clusters but the difference was not significant. This might be due to a small number of cases. Reasons for low incidence and lack of association with known risk factors may be a better literacy rate among mothers and better immunization coverage among children. The recent inclusion of pentavalent vaccine in National immunization schedule will further reduce the morbidity and mortality due to pneumonia.

## Abbreviations

ARI: Acute Respiratory Infections
ASHA: Accredited Social Health Activist
AWW: Anganwadi worker
CI: Confidence Interval
EBF: Exclusive Breast Feeding
FS: Field Supervisor
Hib: Hemophilus Influenza-B vaccine
LPG: Liquified Petroleum Gas
PCV: Pneumococcal Conjugate Vaccine
PHC: Primary Health Center
SD: Standard Deviation
SI: Site Investigator
SPSS: Statistical Package for the Social Sciences
UNICEF: United Nations Children’s Fund
WHO: World Health Organisation
